# Motivational Spiral Models (MSM): common and distinct motivations in context

**DOI:** 10.1186/2193-1801-2-565

**Published:** 2013-10-25

**Authors:** Laurel J Fisher

**Affiliations:** Watervale Systems, PO Box 318, Potts Point, NSW 1335 Australia

## Abstract

Motivational Spiral Models (MSM) show links over time among self concepts, feelings, strategies, skills and participation in everyday activities. In theory, MSM have many common features, with distinct features in particular contexts. This project examined children’s motivation to participate in literacy (MSM-L), social (MSM-S) and physical activities (MSM-P). The participants in Study 1 (*N* = 32) were 9 to 11 years old, and in Study 2 (*N* = 73) were 4 to 12 year old children. Locations were close to the Australian national average in socio-economic indicators, and initial screening showed these were representative samples. Analyses used variable-oriented correlational models as well as person-oriented clusters that suggest the standard and alternative motivational pathways. The results of Study 1 suggested bi-directional links between children’s self concepts and participation in activities. Study 2 identified the common features as: openness and stability over time; and self concepts that motivate and justify participation in activities. Distinct features of MSM-L show the few negative feelings that may limit reading. In MSM-S, self concepts support the positive feelings, and in MSM-P, positive feelings support the task strategies. In conclusion, findings support MSM theory with common features based on self concepts and distinct features of developing motivations in particular contexts. MSM provide a sound base for future research in the contexts of everyday activities for children. In addition, there are practical applications of the findings to prevention, monitoring and intervention programmes.

**Electronic supplementary material:**

The online version of this article (doi:10.1186/2193-1801-2-565) contains supplementary material, which is available to authorized users.

Many local and international programmes for children’s health and well-being provide opportunities for children to engage in everyday activities. Such programmes may focus on children’s motivations to be physically active, develop socially and engage in learning, particularly literacy (e.g., OECD 2002; 2011; see also Lokan et al. 2001 and Vanderstaay 2006). The main aim of this project was to establish Motivational Spiral Models (MSM) as a sound base for children’s motivation to participation in such everyday activities. In principle, MSM address two main challenges in this field. The first challenge is to identify common features of developing motivations that apply across diverse contexts. The second challenge is to identify features of motivations that are distinct to each context; in this project, children’s participation in literacy, social and physical activities. A standard ‘variable-oriented approach’ to MSM examines links among motivational factors over time. For instance, MSM include the links among self concepts and participation in activities. In addition, a so-called ‘person-oriented approach’ uses cluster analysis to explore the diversity in these motivations among particular groups of children (see also Aunola et al. 2002).

## Models of developing motivations

Developing motivations typically consider increases, stability and decreases over time in the factors that support and constrain children’s participation in everyday activities. It is important to consider the evidence carefully. For instance, some phenomena appear to be stable over time (e.g., skills may increase over time although the people are in similar rank orders from one occasion to the next). Other patterns would suggest the phenomena are more open to people’s experiences. This means the rank order may vary over time.

**Figure 1:**
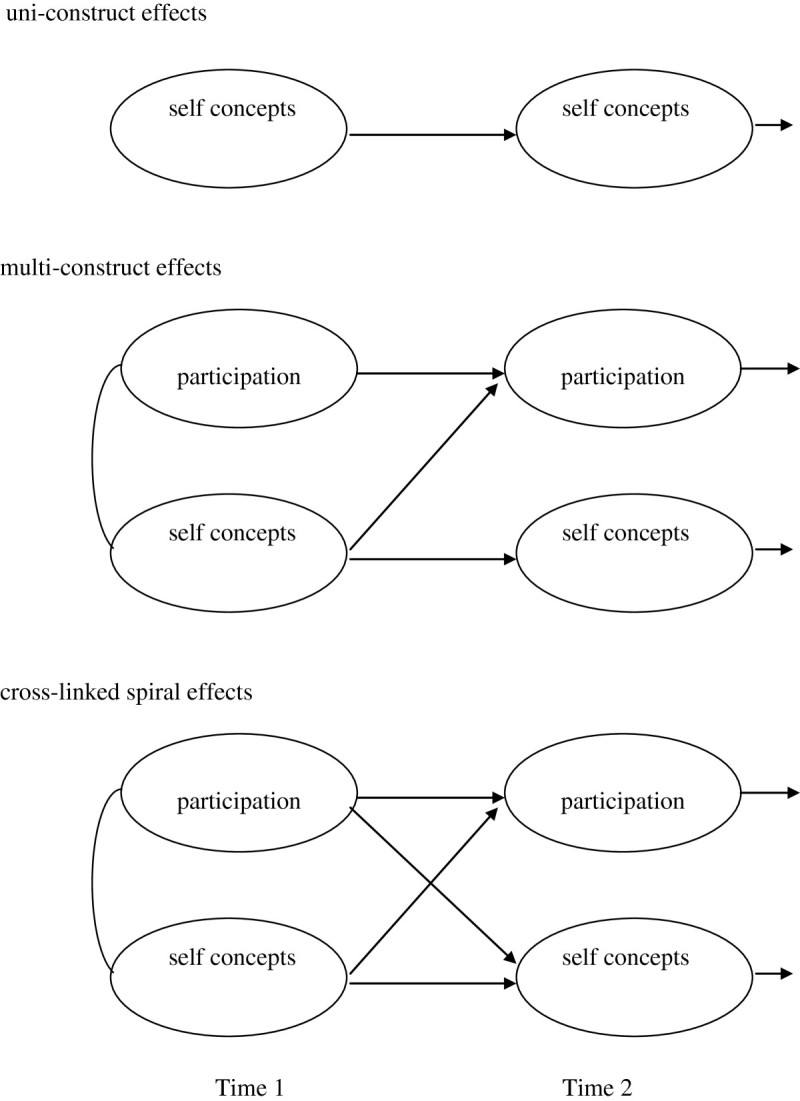
**Proposed links in developing self concepts and participation in activities, over time.**

Given this complexity, models of developing motivations consider several influences among phenomena (see Aunola et al. 2002; Bornholt and Piccolo 2005). Figure 1 shows three effects that are covered in Motivational Spiral Models. The first is a ‘uni-construct effect’ for one phenomenon over time. In this example, findings would show the stability or openness to experience for self concepts over time. The second ‘multi-construct effect’ shows the influence of one phenomenon on another, over time. In this example, initial self concepts influence subsequent participation in an activity (that is, in addition to initial links and stability and openness to experience). The third diagram of ‘cross-linked spiral effects’ shows where phenomena influence each other, over time. In this example, participation in an activity influences subsequent self concepts, and the initial self concepts would also influence subsequent participation in the activity.

The models in Figure 1 are described as ‘uni-construct’ , ‘multi-construct’ and ‘cross-linked spiral effects’. Children’s developing motivations to engage in physical exercise provide prime examples. For instance, a uni-construct effect would show children’s self concepts of physical exercise as moderately stable over time; remaining somewhat open to experience. A multi-construct effect would show children’s self concepts influencing subsequent participation in activities, and cross-linked spiral effects would also show that participation in physical activities support developing self concepts of physical activities. Motivational Spiral Models expand on these models to consider initial and developing links over time among children’s self concepts, positive and negative feelings, strategies, skills and participation in everyday activities.

### Outline of the project

The main proposal of MSM Theory is that developing motivations have common and distinct features across contexts. This proposal is examined for MSM about participation in literacy, social and physical activities. The focus of MSM on children’s participation in context, and the inclusion of feelings and strategies in the model, extend contemporary motivational models and theories. In particular, proposed MSM about literacy extend substantial motivational research with adolescents and models of children’s self concepts and literacy skills (e.g., Aunola et al. 2002; Helmke and Van Aken 1995; Martin 2007; Retelsdorf et al. 2011). In considering MSM about social activities, systematic studies identify critical outcomes of children’s social behaviours (e.g., Prior et al. 2001; Rowe and Rowe 1992; Spira and Fischel 2005). Yet few studies examine children’s motivations for social participation (Malti et al. 2009; Wentzel et al. 2007). The proposed MSM about physical activities also contribute to models of developing motivations by extending clinical and other studies of nutrition, exercise and weight with children and adolescents (e.g., Bornholt and Piccolo 2005; Bjornelv et al. 2011; Chatzisarantis et al. 1998; Downs et al. 2007; Iannotti et al. 2013; Larkin and Hoare 1992; Rose and Larkin 2002).

### Stability over time and responsiveness to experience

Research to date suggests that uni-construct models would show more or less stability as openness to experience for each component in MSM (Aunola et al. 2002; Bornholt and Piccolo 2005). For instance, children’s skills and associated task strategies would be relatively stable over time. For example, Spencer et al. (2003) show stable indicators of cognitive skills over two-week to twelve-week intervals (where correlations *r*_(T1-T2)_ range from 0.8 to 0.9). In contrast, particular feelings about activities are typically fleeting experiences (see Greifeneder et al. 2011; Lewis et al. 2008), meaning that children’s feelings are unlikely to be sustained over time.

The research also suggests openness as moderate stability for participation and self concepts. There are typically variations with time intervals as well as variations that are inherent in personal and social self-categorizations (e.g., Bornholt 2005a; Spencer and Bornholt (2003). Overall, it was expected that there would be variations in uni-construct effects for components of MSM of children’s participation in literacy, social and physical activities.

### Strategic links in the model

#### Self concepts and behaviour

Substantial research identifies a perceptual basis rather than an actual basis to self concepts. In particular, attitude-behaviour models across activities consistently link self concepts with intentions, participation and behavioural choices (e.g., Bornholt 2004; Bornholt and Piccolo 2005; Jacobs et al. 2003; Wigfield and Eccles 2000). Although some studies may report on weak links with large samples, meta analysis and studies in specific contexts (Aunola et al. 2002; Bornholt 2005a; Hattie 1992; 2002; Spencer et al. 2003) show few direct links developing over time between self concepts and standard assessments (e.g., *r* about 0.22 in meta analysis by Hattie 1992). In contrast, consistent weak to moderate links between self concepts and participation in activities are considered here as multi-construct and cross-linked spiral effects. In particular, it is proposed that children’s justifications of participation in activities play a role in developing subsequent self concepts about activities. In addition, it was expected that children’s self concepts about activities would support and constrain subsequent participation in activities. Together, the effects generate proposed spiralling cross-links in the developing motivations for literacy, social and physical activities.

#### Roles of task strategies

Observations of children’s behaviour include task-avoidance and engagement strategies to support developing skills, specifically in literacy (e.g., Bornholt 2002a; Mägi et al. 2013; Paris and van Kraayenoord 1998; Tunmer and Chapman 2002). These task strategies combine observations such as procedural initiative, the use of materials, involvement, pace and task completion. It is plausible that children’s experience of these effective task strategies may be associated with initial positive feelings and limit any negative feelings about the activity. However, the fleeting nature of feelings about activities would suggest that such effects are unlikely to have an impact over time.

It was therefore expected that task strategies in MSM would support initial and subsequent skills, and that children’s experiences of effective task strategies may also support developing self concepts and subsequent participation in the activities.

#### Feelings and self concepts

Feelings are ephemeral ‘affective experiences’ rather than enduring moods (e.g., Greifeneder et al. 2011; Lewis et al. 2008), that are more complex than an unpleasant-pleasant dimension (e.g., Skinner et al. 2009). Instead, models of feelings about activities cover positive feelings (such as feeling alright and pleased) and negative feelings such as worry, guilt, disgust and anger (e.g., Bornholt et al. 2005; Goetz et al. 2012; Greenwald et al. 2000; Niedenthal and Halbertstadt 2000).

In brief, positive and negative feelings may support or constrain initial behaviours. Yet overall effects are unlikely to have an enduring impact on subsequent self concepts, strategies and skills in models of literacy, social and physical motivation. It is also important to consider contemporary research that identifies situations in which specific feelings may alter self judgements, such as ambiguous situations needing elaboration (e.g., Forgas 2001; Gramzow et al. 2000; Greifeneder et al. 2011; Sedikides 1995).

### Motivational Spiral Models–MSM in context

It was proposed that developing motivations for children’s participation have common and distinct features across literacy, social and physical activities. It was expected that:

- self concepts are common features of literacy, social and physical motivations;
- common variations in uni-construct effects include stable skills, moderately stable to more openness in self concepts, strategies and participation, and ephemeral feelings;
- multi-construct and cross-linked spiral effects identify developing motivations among self concepts, positive and negative feelings, strategies, skills and participation; and
- particular positive and negative feelings identify the distinct models of motivation.

## Method

### Design

The main correlational models over time identify MSM in contexts of literacy, social and physical activities. Models in Study 1 link children’s self concepts and participation at Time 1 to Time 3 (a month apart). In Study 2, the MSM link children’s self concepts, feelings, task strategies, skills and participation, at Time 1 and Time 2 (a year apart). The project includes reports of initial screening of general emotional, cognitive, social and physical characteristics in order to ensure these are representative samples of children.

### Participants

The project was located in urban areas close the Australia national average of 1000 in socio-economic indicators (SEIFA, ABS 2006). In Study 1, the participants (*N* = 32) were 9 to 11 years old (mean 9.7, sd 0.5, boys 41%, girls 59%). All the children speak English at school, and most of the children speak English at home (75%) or English with community languages. The participants in Study 2 (*N* = 73) were 4 to 12 years old (mean 8.8, sd 2.4, girls 44%, boys 56%). The children all speak English at school, and English (78%) or English with community languages at home. The retention rate was satisfactory (84%) considering the number families who move house during the year.

### Materials in initial screening

Screening relied on standard assessments that are specifically designed for children. Emotional indicators of general mood used reverse scoring from the Children’s Depression Inventory-Short Form, CDI-S (Kovacs 1992; 1996). The 10 CDI-S items form a reliable indicator (alpha = 0.78). Administration takes less than ten minutes, where the researcher reads each item aloud and the child marks the responses. The scores (0, 1, 2) are added to form scores that range from (0) low to (20) high.

General cognitive screening with SYSTEMS-R (Bornholt et al. 2004; 2010; Fisher et al. 2010; Ouvrier et al. 1999) is reliable (alpha ≥ 0.7) and has strong concurrent validity with full assessments (Differential Abilities Scales DAS, Elliott 2007, GCA *r* = 0.81; Stanford-Binet *r* = 0.88). Individual administration takes under 10 minutes and responses (0/1) to 40 items are summed to form total scores that were expressed as percentages.

General physical indicators used the Body Mass Index (BMI) that is calculated from measured height and weight [kg/m^2^]. The children’s heights and weights were measured in private by a researcher. It is important to note that indicators of BMI are adjusted for age based on WHO charts for children (e.g., de Onis et al. 2007).

### Materials for the components of MSM

#### Self concepts

The ASK-KIDS Inventory (Bornholt [Bibr CR8][Bibr CR9]) is used in clinical and education settings (Bornholt 2004; Marsh et al. 2005; Penn et al. 2001; Russell et al. 2002). Self concepts average the responses to five questions (e.g., ‘How good are you at [the activity]?’). Responses use dot-point ratings, and create reliable self concepts (alpha ≥ .7). Scores range from 1(low) to 5 (high).

Feelings about activities include positive and negative feelings (Bornholt 2002b; Bornholt et al. 2005; Russell 1980). Brief guided conversations about simple line drawings define the activity. Children are interviewed by a trained researcher saying ‘How do you feel doing [the activity]?’. The interviewer reads the 16 words slowly and the child ticks words (or not) to indicate feelings (e.g., ‘alright’ , ‘pleased’ , ‘worried’). Reactive rather than spontaneous techniques overcome any limits in naming feelings. Responses (coded 0/1) form reliable indicators (alpha about 0.70, range 0.62 to 0.87) of positive feelings and related-yet-discrete negative feelings (guilt, worry, disgust and anger) about each activity. The scores are scaled to range from (1) low to (5) high.

#### Participation

Children indicate engagement in activities during interviews by a trained researcher. Simple line drawings define the activity. The example used is reading, that was repeated for social and physical activities. For instance, the interviewer shows the child line drawings of an open book, letters and words on a page, and says ‘Here are some activities for you to do. This activity is about reading. How much do you read?’

Earlier findings used choices among tasks and activities to confirm validity of these responses, yet such choice behaviours are necessarily limited by dependent outcomes as 1^st^ 2^nd^ or 3^rd^ choice (see Coleman and Bornholt 2003; Bornholt 2004). The direct items used here are ideal where children are about to engage in activities, which ensures that the context heightens the salience. Interviews took place in the school hall where children were engaged in cognitive, social and physical assessments as part of the project. The responses used five dot-point ratings from (1) low to (5) high.

Task strategies combine procedural initiative, sequences, persistence, involvement and pace (Bornholt 2002a; Paris & van Kraayenoord 1998). Strategies were observed during standard assessment tasks. Observations form reliable indicators (alpha ≥ 0.70, inter-rater reliability *r*_(AB)_ = 0.75). The scores range from (1) low to (5) high.

Literacy, physical and social skills used standard assessment materials. Indicators of literacy skills used reliable and valid TORCH Test of Reading Comprehension (ACER 2003). TORCH is Rasch-modelled so that children may read various age-appropriate passages and the scores can be related to an underlying TORCH scale. Administration of TORCH materials may be with individual children or in small groups.

Indicators of physical skills used the Movement Assessment Battery for Children (Henderson and Sugden 1992; Schulz et al. 2011). Eight items in age bands include reversed coded manual dexterity, ball and balance skills (Brake and Bornholt 2002,2004; Bornholt and Piccolo 2005) as a reliable scale (alpha ≥0.7) from (1) low to (5) high.

Social behaviours were indicated by the RBRI Rowe Behaviour Rating Inventory (Rowe and Rowe 1995; Fisher and Spencer 2013). Children were observed and filmed during open-ended construction tasks for small groups of four or five children. Coding used the 12-item RBRI teacher rating form. Ratings form reliable scales (alpha = 0.82 to 0.91, inter-rater reliability *r*_(AB)_ = 0.70). The scores range from (1) low to (5) high.

### Procedure

The project was approved by the University Ethics Committee and also by the State Government Department of Education. To control for broad social factors, the location was selected carefully. The government co-educational schools are as close as possible to the Australian national average in socio-economic indices (SEIFA, ABS 2006).

Administrators posted information and consent forms to parents or guardians. In co-operation with teachers, and written parental consent, children were interviewed and assessed by trained researchers individually and small groups in the school hall. With various starting points, children moved among activities, with rests, over 90 minutes.

#### Analyses

Descriptive (mean, SD), inferential statistics (t-tests, ANOVA, correlation, regression, with standard criteria, p < .05) and cluster (k-means) used SPSS Windows. Cluster analysis is an exploratory technique to group similar individuals systematically, with visual aids to interpret outcomes (e.g., Everitt and Dunn 1983; Kanungo et al. 2002).

## Results

### Study 1 developing self concepts and participation

The initial screening for participants in Study 1 included emotional, cognitive and physical indicators. The findings suggest these children were a representative sample where profiles were similar to population norms, considering the age of the children. Profiles show BMI (mean 18.9 SD 3.7, de Onis et al. 2007), reading skills (TORCH mean 34.5, SD 8.6 (ACER 2003) and mood (mean 4.5, SD 4.3) indicated few depressive symptoms (Kovacs 1996). Preliminary analysis showed that the profiles were similar for girls and boys (t-tests were not statistically significant, ns). In addition, there were few effects of age, where regression coefficients were not statistically significant, except for self concepts about social activities (β = 0.34, p < .05) due to variations in mood.

The main results of Study 1 show developments in self concepts in relation to participation over time (on three occasions, a month apart). Results in Table 1 show positive self concepts and participation in literacy, social and physical activities.

**Table 1:** **Profiles and development of self concepts and participation over time (a month apart)**

Context	Means (sd)	Time 1	Time 2	Time 3
Literacy	self concepts	3.88 (0.89)	4.05 (0.83)	4.03 (0.91)
	participation	4.22 (0.83)	3.89 (1.18)	4.04 (1.07)
Social	self concepts	4.13 (1.02)	4.21 (0.92)	4.13 (0.96)
	participation	4.25 (1.02)	4.30 (0.99)	3.92 (1.36)
Physical	self concepts	3.93 (1.22)	4.47 (0.61)	4.31 (1.03)
	participation	4.34 (1.15)	4.52 (0.87)	4.21 (1.07)
**Context**	**Regression (β) p < .05**		**Time (1 to 2)**	**Time (2 to 3)**
Literacy	self concepts		0.32	0.27
	participation		ns	0.52
	self concepts: participation		ns	0.25
	participation: self concepts		0.38	0.20
Social	self concepts		0.86	0.58
	participation		0.51	0.33
	self concepts: participation		ns	0.48
	participation: self concepts		ns	0.28
Physical	self concepts		0.25	0.57
	participation		0.52	0.36
	self concepts: participation		ns	0.35
	participation: self concepts		0.35	0.25

**Figure 2:**
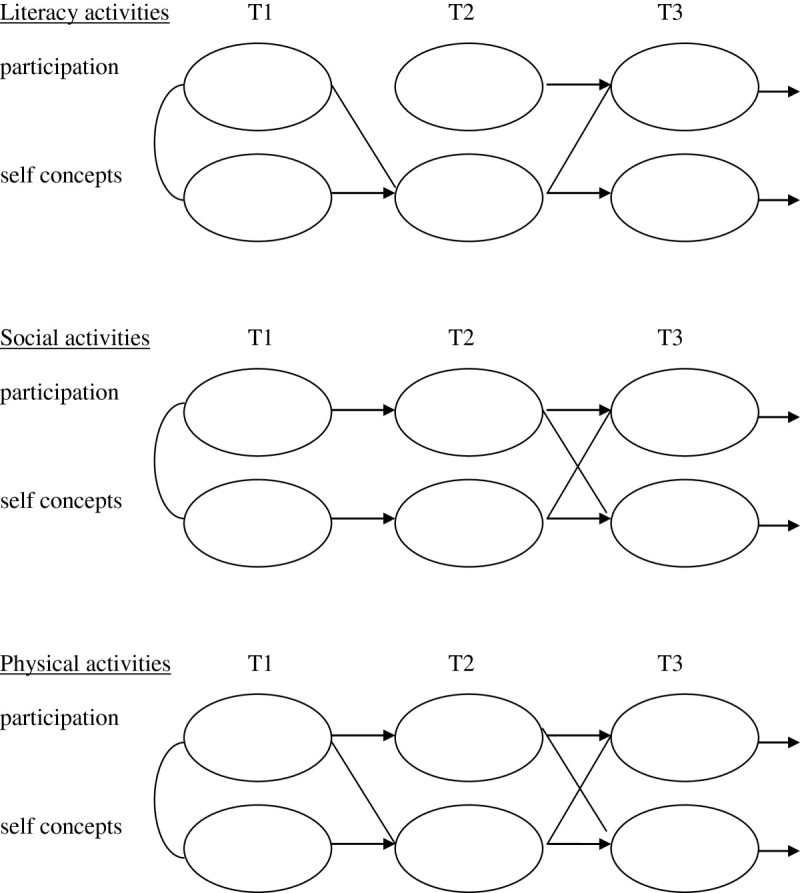
**Conceptual models of self concepts and participation over time (T1 to T3, a month apart).** Notes. Links are significant initial correlations (p < .05) and regression coefficients (p < .05).

Figure 2 shows significant links in the conceptual models (for details see Table 1). The ‘uni-construct’ links from one occasion to the next indicate openness to experience. ‘Multi-constructs’ from self concepts to participation, and participation to self concepts, show additional influences over time. Spiral effects are suggested for literacy activities, from Time (1 to 2), and Time (2 to 3), and are evident from Time (2 to 3) as cross-links between self concepts and participation in social and physical activities.

In summary, the findings suggest developing motivations as links among self concepts and participation in the contexts of literacy, social and physical activities. In particular, findings suggest that children’s self concepts may motivate and justify participation. Overall, the results support careful screening, particularly for mood, and suggest that it is worthwhile tracing developing motivation over longer time intervals in Study 2.

### Study 2 MSM in context–part 1 screening

Screening of general emotional, cognitive, social and physical indicators confirm the representative sample, to suggest it is appropriate to draw inference from the findings. Specifically, profiles were close to 50th percentile of BMI from measured heights and weights (WHO charts by age, *t*; = 0.62 ns, see de Onis et al. 2007). There was some variation in positive moods (CDI-S 0 to 14, mean 3.7, SD 4.1), and no depressive symptoms (Kovacs 1992). Cognitive screening was close to population means by age (t-tests ns, with one exception for seven year old children, *t* = 4.5, p < .05, Bornholt et al. 2004), and movement skills were above the 50th percentile (*t* = 6.6, p < .001, effect 0.7 SD, Henderson and Sugden 1992). Children’s social behaviours were close to the 50^th^ percentiles for observed sociable (*t* = 0.3, ns), attentive (*t* = 5.3, p < .001, effect 0.4 SD) and settled behaviours (*t* = −1.3, ns, see Fisher and Spencer 2013; Rowe and Rowe 1995).

### Results for MSM motivational spiral models

#### Profiles by age and gender

**Figure 3:**
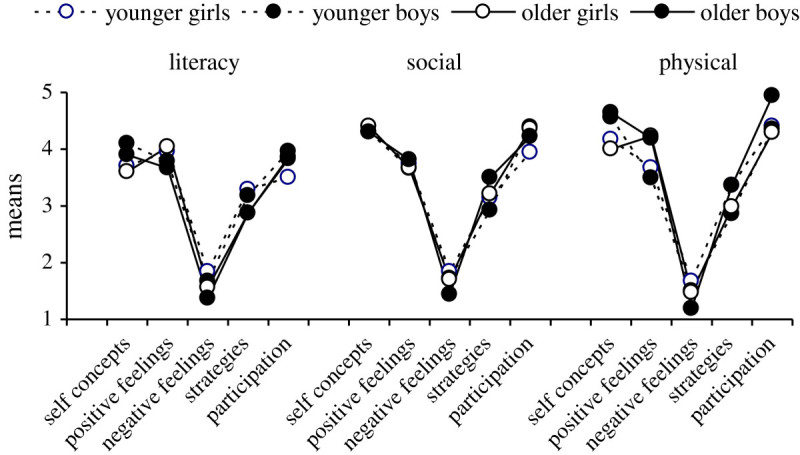
**Profiles of self concepts, positive and negative feelings, task strategies and participation in literacy, social and physical activities at Time 1, for younger and older girls and boys.** Notes. a. Profiles were similar for younger and older girls and boys (F-tests (df 3,69) ns), with one exception that physical self concepts were higher for boys than girls (0.56 SD). b. Age had no influence (range in β from −0.21 to 0.27) with two exceptions for social strategies (β = 0.34, p < .05) and feeling positive about movement (β = 0.37, p < .05).

Profiles in Figure 3 were similar for younger and older girls and boys in self concepts, feelings, strategies and participation (F-tests were not statistically significant, with few exceptions). As standard assessments, children’s skills were unbiased by gender (literacy *F* = 1.4 ns, social *F* = 2.8 ns, physical *F* = .30 ns) and skills tended to increase with age (literacy β = 0.56, social β = 0.23, physical β = 0.30). Therefore, subsequent analyses used age-based percentiles of skills, and the responses for younger and older girls and boys were combined.

#### Profiles of motivations and behaviours

The profiles in Table 2 show the reliability of materials (alpha about 0.7). On average, the profiles for Time 1 and Time 2 describe the children’s moderate self concepts, positive feelings with few negative feelings, moderate strategies, skills and participation in literacy, social and physical activities.

**Table 2:** **Profiles for Motivational Spiral Models of Literacy, Social and Physical activities, with reliability, mean (sd) and range, at Time 1 and Time 2 (a year later)**

Motivational Spiral Models		Time 1			Time 2	
		Alpha	Mean	(sd)	Mean	(sd)
MSM-L Literacy	skills ^a^	.79	3.1	(0.9)	3.3	(0.7)
	task strategies	.73	3.2	(0.9)	3.4	(1.0)
	self concepts	.86	3.8	(1.1)	3.9	(1.1)
	positive feelings	.69	3.7	(1.6)	3.3	(1.3)
	negative feelings	.83	1.6	(0.9)	1.3	(0.5)
	participation	.77	3.7	(1.5)	3.7	(1.4)
MSM-S Social	skills ^a^	.90	4.0	(0.9)	4.7	(0.6)
	task strategies	.83	3.1	(1.0)	3.4	(0.9)
	self concepts	.68	4.3	(0.7)	4.2	(0.9)
	positive feelings	.67	3.6	(1.5)	3.1	(1.3)
	negative feelings	.66	1.7	(0.8)	1.4	(0.5)
	participation	.71	4.2	(1.2)	4.4	(1.0)
MSM-P Physical	skills ^a^	.70	3.8	(0.7)	4.2	(0.9)
	task strategies	.83	3.1	(1.1)	3.5	(0.9)
	self concepts	.86	4.4	(0.9)	4.3	(0.8)
	positive feelings	.71	3.8	(1.4)	3.6	(1.2)
	negative feelings	.90	1.4	(0.9)	1.2	(0.5)
	participation	.77	4.5	(0.9)	4.4	(1.1)

Note. ^a^ for ease of comparison the skills scores are re-scaled from (1) low to (5) high.

Self concepts at Time 1 were within an optimal range for everyday activities. That is, on average, optimal self concepts are somewhat above the mid-point of scales; about 3.8 on five-point scales (see Bornholt 2005a). On average, children also express moderately positive feelings with little guilt, anger, disgust and few worries about activities. Table 2 also shows moderate task strategies with observations that were close to the mid-points of scales for literacy, social and physical activities (t-tests were not significant). In addition, children’s participation was close to optimal ratings of 3.8 in literacy (*t* = 1.6 ns). Participation was somewhat stronger in social activities (*t* = 5.2 p < .001, effect size 0.4 SD) and also for children’s participation in physical activities (*t* = 10.0 p < .001, effect size 0.5 SD).

### Study 2 MSM in context–part 2. Common and distinct features

**Figure 4:**
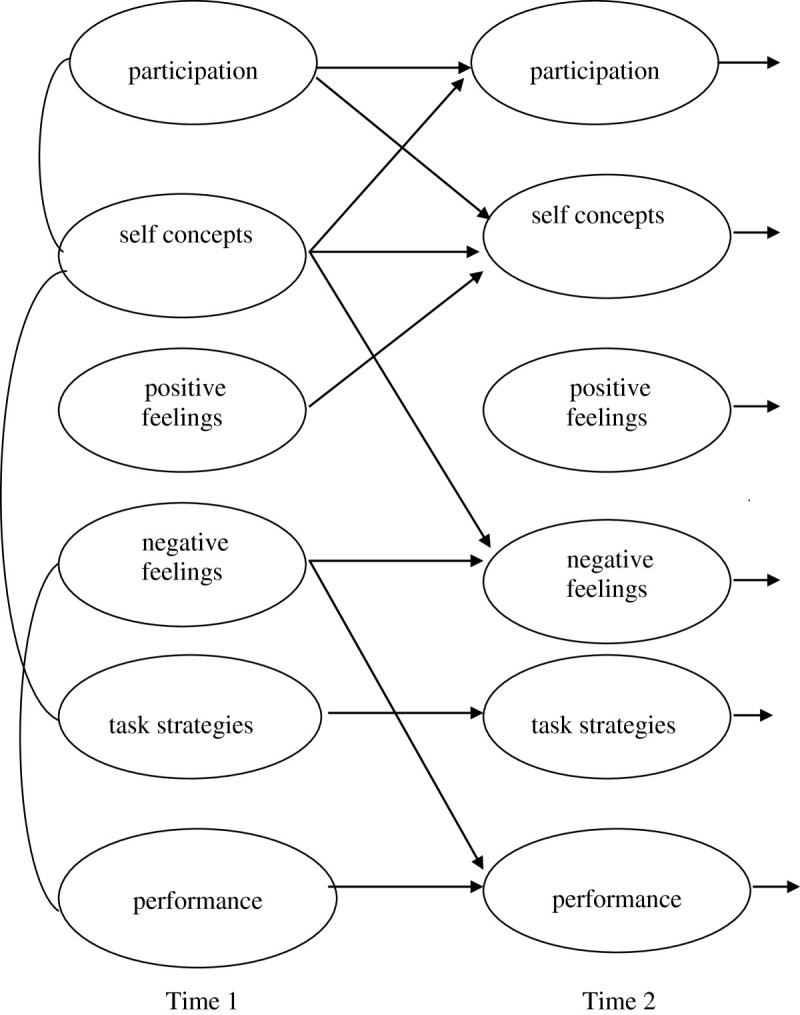
**Conceptual model of Motivational Spiral Model-Literacy (MSM-L) with significant initial correlations at Time 1 and significant paths from Time 1 to Time 2 (a year later).**

**Figure 5:**
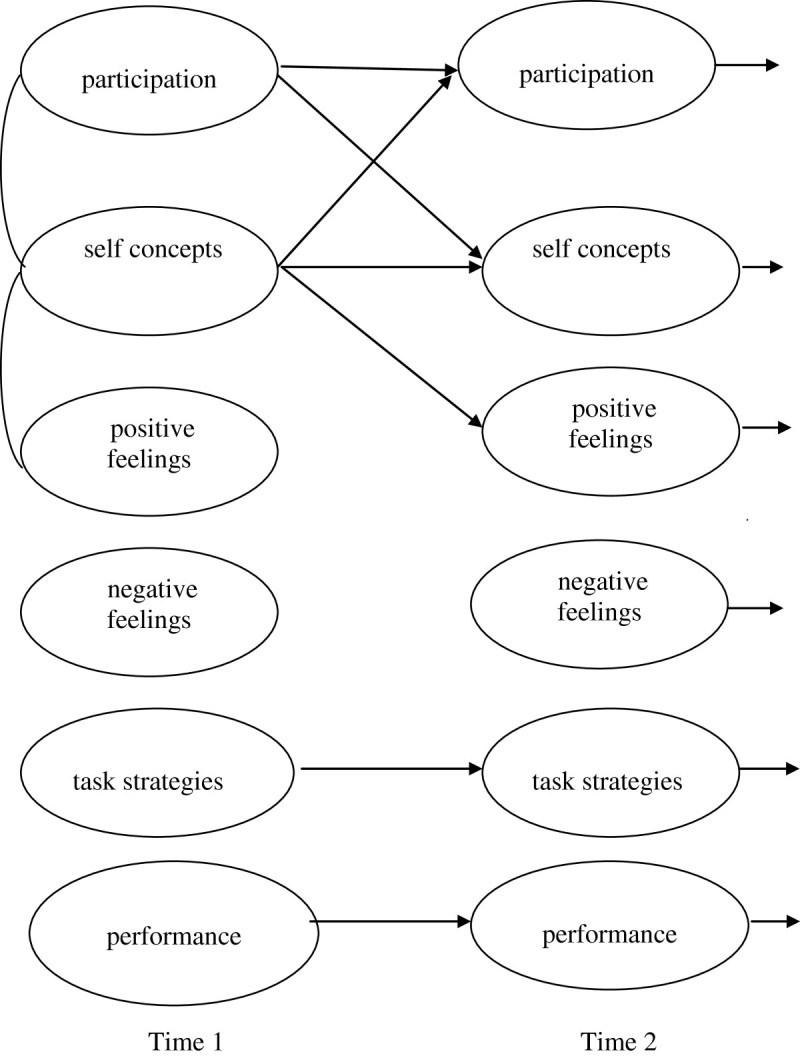
**Conceptual model of Motivational Spiral Model -Social (MSM-S) with initial significant correlations at Time 1 and significant paths from Time 1 to Time 2 (a year later).**

**Figure 6:**
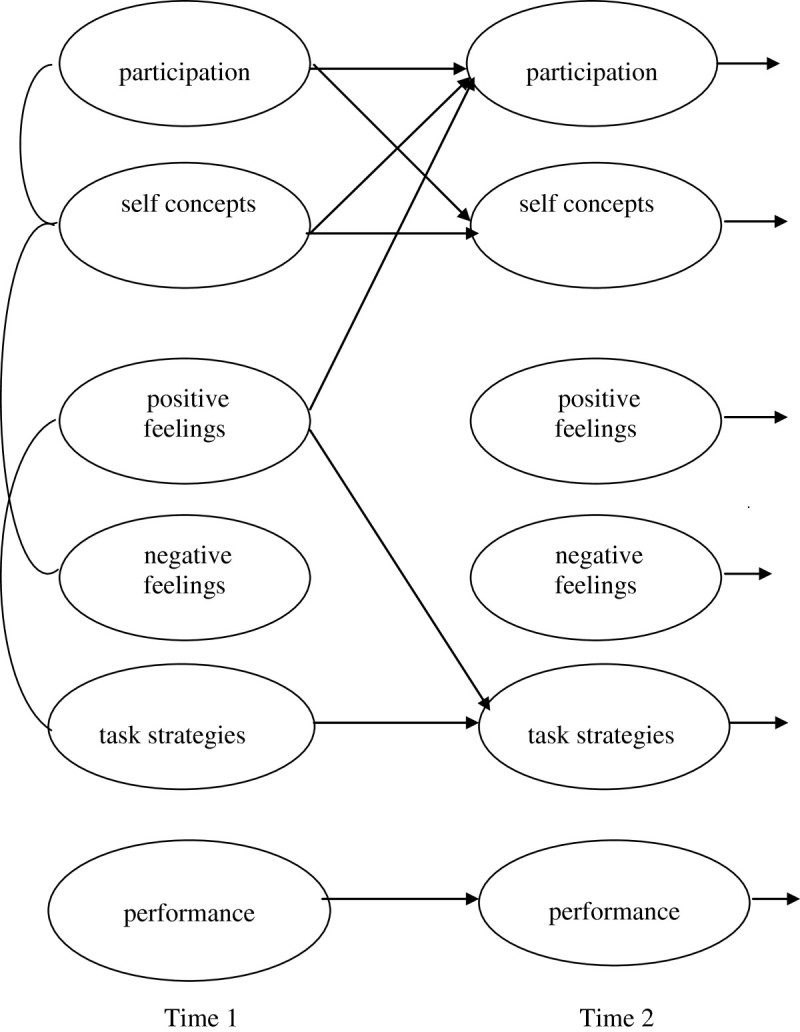
**Conceptual model of Motivational Spiral Model - Physical (MSM-P) with significant correlations at Time 1 and significant paths from Time 1 to Time 2 (a year later).**

Part 2 of the study was designed to examine common and distinct features of MSM about literacy, social and physical activities. Figure 4 describes children’s developing motivations to participate in literacy activities (MSM-L). Figure 5 describes motivations to participate in social activities (MSM-S), and Figure 6 describes motivations for physical activities (MSM-P). Results for each MSM show significant links (*r*, p < .05) among initial components at Time 1, stability and openness to experience over time (β T1-T2), and strategic links and the spiralling motivations over time (β T1-T2, p < .05).

Results show two common features of developing motivations in MSM-L, MSM-S and MSM-P. The common features were: (a) the uni-construct stability and openness to experience; and (b) the cross-linked spiral effects on self concepts and participation.

### Common features of developing motivations

#### Stability and openness to experience

The results show that skills are generally stable about literacy and physical activities (MSM-L β_(T1-T2)_ = 0.82, MSM-P β_(T1-T2)_ = 0.80). Social skills show moderate stability, tending to be more open to experience from one occasion to the next (MSM-S β_(T1-T2)_ = 0.32).

Other components are moderately stable, remaining somewhat open to experience. Self concepts are typically open to experience (MSM-L β_(T1-T2)_ = 0.57, MSM-S β_(T1-T2)_ = 0.34 and MSM-P β_(T1-T2)_ = 0.34). Task strategies are also open to experience (MSM-L β_(T1-T2)_ = 0.44, MSM-S β_(T1-T2)_ = 0.53 and MSM-P β_(T1-T2)_ = 0.50). The indicators of children’s participation show variations in stability and openness to experience (MSM-L β_(T1-T2)_ = 0.22, MSM-S β_(T1-T2)_ = 0.51,MSM-P β_(T1-T2)_ = 0.33).

In contrast, children’s positive feelings about activities tend to be ephemeral (MSM-L β_(T1-T2)_ = 0.04, MSM-S β_(T1-T2)_ = 0.14, MSM-P β_(T1-T2)_ = 0.13). Negative feelings also tend to be ephemeral although negative feelings about reading show some stability over a year (MSM = R β_(T1-T2)_ = 0.38, MSM-S β_(T1-T2)_ = 0.19, MSM-P β_(T1-T2)_ = 0.13).

#### Cross-linked spiral models

The results show that self concepts have a consistent role in children’s developing motivations for participation. Initial self concepts are associated with participation in literacy, social and physical activities (MSM-L *r*_(T1)_ = 0.32, MSM-S *r*_(T1)_ = 0.49, MSM-P *r*_(T1)_ = 0.30). The cross-links show that self concepts feed forward to motivate subsequent participation (MSM-L β_(T1-T2)_ = 0.24, MSM-S β_(T1-T2)_ = 0.30, MSM-P β_(T1-T2)_ = 0.26). In addition, these cross-links show that justifications of participation tend to support subsequent self concepts in literacy, social and physical activities (MSM-L β_(T1-T2)_ = 0.32, MSM-S (β_(T1-T2)_ = 0.21, MSM-P β_(T1-T2)_ = 0.25).

### Distinct features of developing motivations

This part of the results identifies the distinct features about children’s developing motivations in the contexts of literacy, social and physical activities. The focus is on the role of feelings in initial motivations, and related multi-construct effects in each context.

#### MSM-L literacy

Figure 4 shows initial motivations where negative feelings tend to limit literacy skills, specifically for children who worry (*r* = 0.25, p < .05). Over time, children’s positive feelings support self concepts (MSM-L β_(T1-T2)_ = 0.43), limit negative feelings (MSM-L β_(T1-T2)_ = −0.24) and subsequent skills (MSM-L β_(T1-T2)_ = −0.33).

#### MSM-S social

The results for MSM-S in Figure 5 show that feelings are distinct features in initial motivations where positive feelings are associated with self concepts about social activities (*r* = 0.32). In addition, self concepts support subsequent positive feelings about social activities (MSM-S β_(T1-T2)_ = 0.23).

#### MSM-P movement

Figure 6 shows that in initial motivations, negative feelings are associated with self concepts (*r* = 3.0) for children feeling guilty or worried, and positive feelings are associated with task strategies (*r* = 3.2). In addition, positive feelings about physical activities support participation in subsequent activities (MSM-P β_(T1-T2)_ = 0.34) and the strategies needed to initiate and complete tasks (MSM-P β_(T1-T2)_ = 0.30).

### Study 2 MSM in context–part 3. Motivational pathways

Common and distinct features of MSM are results from Parts 1 and 2 using standard ‘variable-oriented approaches’ to analysis. In Part 3, developing motivations also benefit from a so-called ‘person-oriented approach’. This approach used exploratory cluster techniques to form groups of similar children. Exploring children’s motivations uses close analysis of profiles. Interpretations of the basic clusters of motivations suggest a standard pathway, as well as an alternative pathway through developing motivations.

The clusters described in Table 3 are interpreted to describe MSM pathways through developing motivations. Interpreting profiles builds deeper understandings of children described by MSM general models. Simple clusters are appropriate with a representative sample. A basic non-hierarchical method suggested two clusters. Cluster A ‘alternative pathways in MSM’ includes some participants in each context (MSM-L 38%, MSM-S 18%, MSM-P 18%). However, most participants are in Cluster B as ‘standard pathways in MSM’ in these everyday contexts (MSM-L 62%, MSM-S 82%, MSM-P 82%).

**Table 3:** **Profiles for clusters** ^**a**^ **of children in MSM pathways (Time 1 to Time 2) for skills, self concepts, feelings, strategies and participation in literacy, social and physical activities**

MSM pathways		Cluster A		Cluster B	
Profiles		Time1	Time2	Time1	Time2
**MSM-L Literacy**		**Cluster A (38%)**		**Cluster B (62%)**	
	skills ^b^	3.1	3.2	3.2	3.4
	task strategies	1.0	1.1	3.8	4.5
	self concepts	1.4	1.8	4.4	4.8
	positive feelings	3.7	2.3	5.0	5.0
	negative feelings	1.7	1.0	5.0	2.0
	participation	1.0	2.0	5.0	5.0
**MSM-S Social**		**Cluster A (18%)**		**Cluster B (82%)**	
	skills ^b^	3.5	4.4	4.0	4.7
	task strategies	3.9	2.5	2.7	3.8
	self concepts	4.0	3.2	4.8	5.0
	positive feelings	2.3	1.0	5.0	5.0
	negative feelings	1.3	1.3	2.0	1.3
	participation	1.0	5.0	1.0	5.0
**MSM-Physical**		**Cluster A (18%)**		**Cluster B (82%)**	
	skills ^b^	3.7	4.0	4.0	4.5
	task strategies	3.5	5.0	1.0	4.2
	self concepts	3.2	2.6	5.0	5.0
	positive feelings	5.0	1.0	2.3	5.0
	negative feelings	5.0	2.3	1.3	1.0
	participation	3.0	1.0	2.0	5.0

Notes:

^a^ Cluster analysis used ‘K-means’ non-hierarchical method (e.g., Aunola et al. 2002).

^b^ For ease of comparison skills scores are re-scaled to range from 1 (low) to 5 (high).

#### Literacy

Alternative pathways in MSM-L (Cluster A) highlight an increase over time in literacy self concepts and reduced negative feelings with an increase in participation over a year. In contrast, standard pathways in MSM-L (Cluster B) describe improved strategies and self concepts, a marked reduction in negative feelings about literacy, with a slight increase in skills and sustained participation in reading activities over the year.

#### Social

Interpretations of alternative pathways in MSM-S (Cluster A) are more interesting for social skills. Profiles in Cluster A highlight the increased social skills although strategies and self concepts decreased somewhat, with a sharp increase in participation. Standard pathways in MSM-S (Cluster B) show increased strategies, reduced negative feelings, increased self concepts and skills, and also a sharp increase in participation over the year.

#### Physical

Alternative pathways for MSM-P (Cluster A) show increased strategies and skills, with reduced self concepts, positive feelings and participation over a year. In contrast, a standard MSM-P pathway (Cluster B) show sustained self concepts, with increases in task strategies, skills, positive feelings and participation in physical activities over the year.

In summary, the clusters identify variations in the motivational pathways that are suggested here for several groups of children. This approach uses detailed information to form groups of similar children, where larger samples may allow cluster programmes to suggest further refinements in the diverse motivational pathways. For this sample of children, the suggested profiles make a useful addition to standard variable-oriented approaches as models of developing motivations. Such a person-oriented approach contributes to an understanding of the diversity in developing motivations in MSM.

## Discussion

The results support the main proposal in identifying the common and distinct features of children’s developing motivations for literacy, social and physical activities. In particular, the common features of MSM developing motivations are evident across contexts. These include proposed variations among MSM components in the stability and openness to experience, and the cross-linked spirals that characterize Motivational Spiral Models. As expected, the features of MSM that are distinct to each context are connected with children’s positive and negative feelings about these everyday activities.

In brief, the findings highlight the common social-cognitive processes as well as the distinct roles of positive and negative feelings in children’s developing motivations in the contexts of literacy, social and physical activities.

### Limitations and strengths in the design

It is important to note features that may limit and strengthen interpretations of the findings. For instance, initial screening identified the representative sample of girls and boys across a wide age range in locations close to the national socio-economic average. It is also a critical feature of the design that materials are appropriate for such intensive fieldwork, without inducing fatigue or boredom. The materials are brief, meaningful and useful indicators of self concepts, feelings, strategies, skills and participation that are appropriate for such typically diverse groups of children (e.g., Bornholt et al. 2005; Marsh et al. 2005; Davis-Kean and Sandler 2001).

The profiles for these children were age-appropriate for the population, with few exceptions. Screening is an essential although time-consuming feature of projects in naturalistic settings. Attention to screening cognitive, physical, social and emotional indicators, and project location, therefore suggests where the findings may be applied.

The intensive fieldwork with individual administration led to one of the main limitations in the small sample size in Study 1. As with any design, there are benefits and weakness in case studies, in small samples on specific factors, and in moderate and large samples. For instance, there may be benefits in MSM over several occasions, say, two or three months apart, although substantial samples would be needed for structural equation modelling and latent growth curves. However, such considerations may be balanced by the depth of findings presented here with representative samples in locations of average socio-economic indicators. In some ways, the sample in Study 1 may be considered adequate for the purpose, where findings underlined the importance of careful screening, and are confirmed with another sample of children in Study 2. In contrast, the sample in Study 2 has sufficient power (83%, r ≥ 0.3, p < .05). Therefore, it is reasonable to conclude that the sample size is satisfactory for the purpose, and that we may draw on these findings with some confidence. It remains for further research to apply the Motivational Spiral Models to additional activities and in other locations.

### Common features of motivational spiral model MSM

Results support the proposed MSM based on four conditions, with few exceptions. First, self concepts are important in children’s initial motivations. At Time 1, self concepts are linked with task strategies in literacy, positive feelings of social activities, and self concepts tend to limit negative feelings about physical activities. In particular, the self concepts are what children think about themselves and these activities that are associated with participation in literacy, social and physical activities, notably over and above skills on standard assessments. Findings confirm correlational and experimental evidence from attitude-behaviour and related models (Bornholt 2005a; Hattie 1992; Wigfield and Eccles 2000). It is evident that self concepts support children’s participation in activities in these diverse everyday contexts.

The second condition is that systematic variations in uni-construct models generally apply across contexts. Specifically, results show the stability of skills in literacy and physical movement. Yet social behaviours are more open to experience, so perhaps these activities are inherently context-dependent (Fisher and Spencer 2013). In brief, for skills that vary systematically with age, the rank order seems to remain stable over time.

As expected, children’s self concepts are somewhat open to experience. This applies across literacy, social and physical activities. The findings confirm that self evaluations draw on personal and social interpretations of events, in a common if controversial finding (see Aunola et al. 2002; Bornholt 2005a; Harter 1996; Retelsdorf et al. 2011; Spencer et al. 2003). Findings also highlight the ephemeral nature of positive and negative feelings that are typically unsustained over time, with notable exceptions.

The third condition in Motivational Spiral Models considers multi-construct and cross-linked spirals from Time 1 to Time 2. For instance, multi-constructs link feelings with other motivations and behaviours, in particular contexts (e.g., Bornholt et al. 2005). MSM are characterised by spiralling cross-links among self concepts and participation. Results clearly show that self concepts motivate and justify participation, and the spiral effects are common to developing motivations for literacy, social and physical activities.

Condition four concerns the positive and negative feelings in developing motivations. Any situations in which feelings are associated with initial self concepts are particularly interesting. The research in other contexts (e.g., Bornholt et al. 2005; Forgas 2001; Greifeneder et al. 2011; Sedikides 1995) suggests that feelings may alter self concepts, in specific situations. The implications are that social-emotional processes in initial motivations (at Time 1) suggest variations of meanings, as perhaps ambiguous situations. Specifically, results show no links for initial self concepts with positive and negative feelings about literacy. In contrast, there are subtle links between positive feelings and self concepts about social activities, and also where guilt or worry may alter self concepts about physical activities. It seems reasonable to conclude that positive and negative feelings about activities tend to elaborate self concepts in these particular situations.

It is evident children generally feel moderately positive and that few children feel guilt, worry, anger or disgust about literacy, social and physical activities. Feelings are brief responses to specific events (e.g., apparent exceptions suggest absence of negative feelings about literacy over a year). Interesting links with motivations and behaviours include positive feelings to task strategies and participation in physical activities, and negative feelings to subsequent reading skills (e.g., moderate initial worry supports an increase in subsequent reading skills, and strong initial concerns by few children limit subsequent literacy skills). Detailed findings identify where case studies and experiments in naturalistic settings would be worthwhile on the roles of feelings in specific contexts. Overall findings show that feelings are typically unsustained over time, and that positive and negative feelings would have quite distinct roles in MSM, in specific situations.

## Conclusion

Understanding the common and distinct features of children’s developing motivations establishes the contribution of Motivational Spiral Models MSM to theories and models of motivation. In addition, the exploration of standard and alternative MSM motivational pathways across contexts extends earlier research on developing motivation and skills that also consider accumulative models in development (e.g., Aunola et al. 2002; Prior 1999). The person-oriented and variable-oriented approaches to understanding children’s developing motivations extend findings in large-scales studies with adolescents, and also correlation and experimental studies with children in specific fields (e.g., Aunola et al. 2002; Bornholt et al. 2005; Chapman et al. 2000; Malti et al. 2009; Martin 2007; Prior 1999; Retelsdorf et al. 2011). The major contribution of this project is to contextualise MSM Theory of developing motivations, by detailed examinations of the common and distinct features of children’s motivations in the contexts of everyday activities.

The proposed Motivational Spiral Models across these diverse contexts open avenues for research on children’s developing motivations to participate in activities, building on earlier work about other activities and motivation in developing skills. In particular, findings confirm and expand on self concepts that motivate and justify engagement in activities, and highlight the standard and alternative pathways that implicate children’s thoughts, feelings, strategies, skills and participation.

Findings provided ample support for common and distinct features of Motivational Spiral Models. It is clear that self concepts are important in motivations for alternative and standard paths to participation in activities. MSM advance traditions of research on social-cognitive processes that support participation in activities, and renews our interest in the justifications of participation that support developing self concepts. It is important to notice the common links in MSM as uni-construct models of stability to highlight the openness to experience. This is where intervention programmes would be effective.

Understanding children’s developing motivations also focuses attention on particular thoughts and feelings. Ongoing projects examine children’s self concepts about the body as foundations for early depressive symptoms, where positive and negative feelings tend to develop somewhat independently (see also Bornholt et al. 2005). Other projects consider the strategic situations that may sustain emotional responses to experience for children with specific clinical conditions, and the roles of parents, teachers and trained observers, as well as self concepts about social behaviours in context (Fisher and Spencer 2013).

The findings from person-oriented and variable-oriented approaches support proposed Motivational Spiral Models, and suggest extending MSM to vary the time intervals in other contexts. There are clear applications to differential rather than one-size-fits-all programmes in clinical and community settings. It is therefore reasonable to conclude that common and distinct features of MSM advance theories of developing motivation for participation in the activities that support general health and well-being.

## Authors’ original submitted files for images

Below are the links to the authors’ original submitted files for images. Authors’ original file for figure 1 Authors’ original file for figure 2 Authors’ original file for figure 3 Authors’ original file for figure 4 Authors’ original file for figure 5 Authors’ original file for figure 6 Authors’ original file for figure 7
