# Supplementary figures and images for: Motivational Spiral Models (MSM): common and distinct motivations in context
Source: Springerplus. 2013 Oct 25;2(1):565. doi: 10.1186/2193-1801-2-565 (PMC3825061; doi:10.1186/2193-1801-2-565)

### uni-construct effects

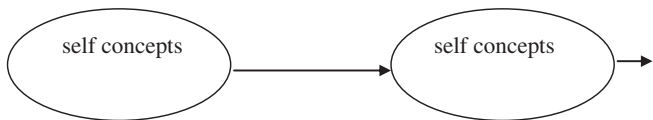

### multi-construct effects

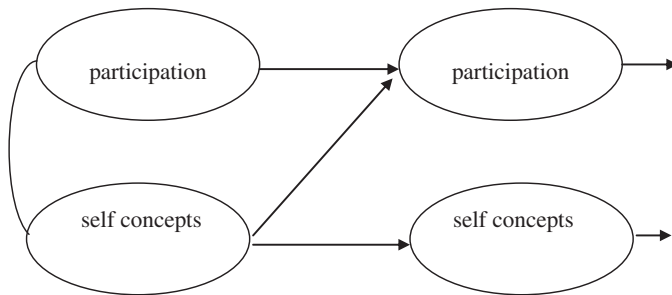

### cross-linked spiral effects

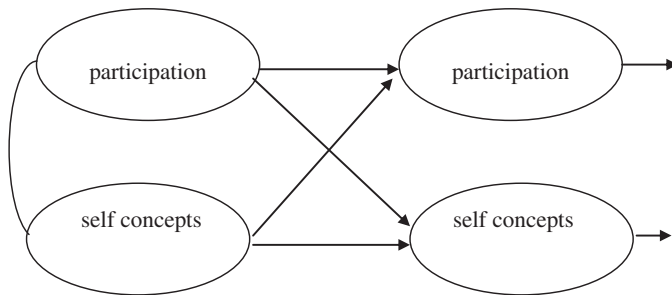

Time 1

Time 2

Supplement: Supplementary file 1 — Authors’ original file for figure 1 [file 40064_2013_619_MOESM1_ESM.pdf]

---○--- younger girls    ---●--- younger boys    —○— older girls    —●— older boys

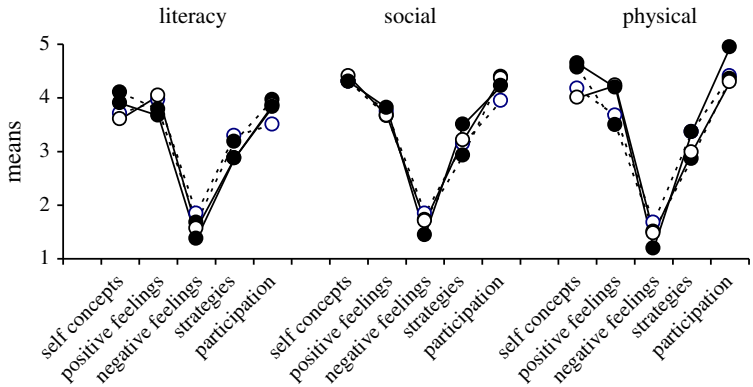

Supplement: Supplementary file 3 — Authors’ original file for figure 3 [file 40064_2013_619_MOESM3_ESM.pdf]

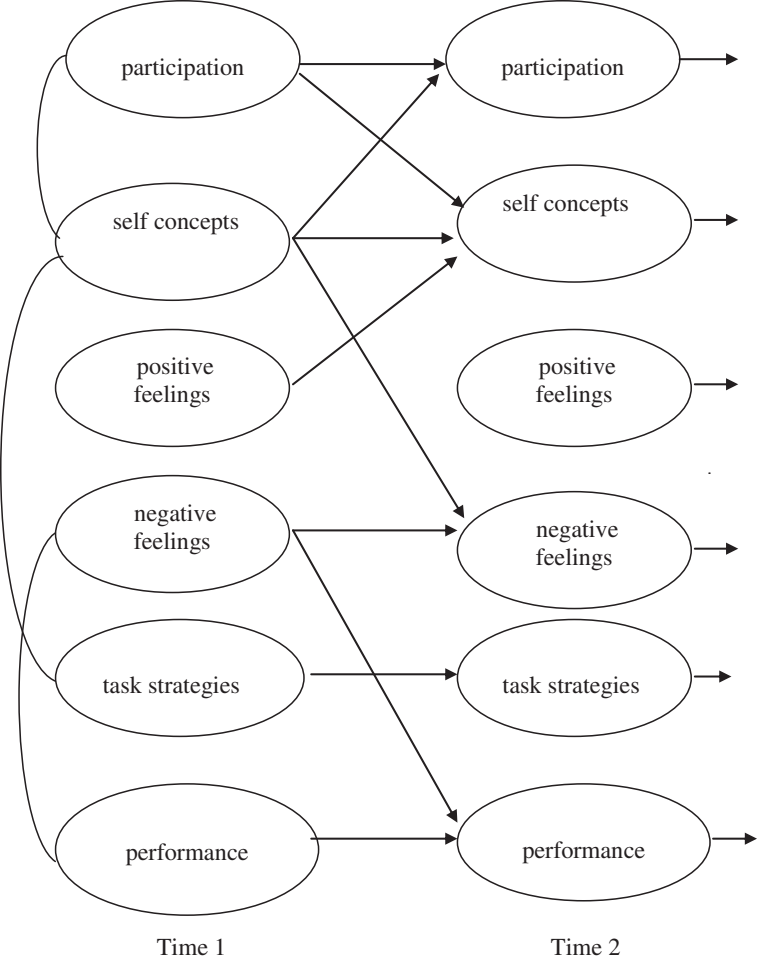

Supplement: Supplementary file 4 — Authors’ original file for figure 4 [file 40064_2013_619_MOESM4_ESM.pdf]

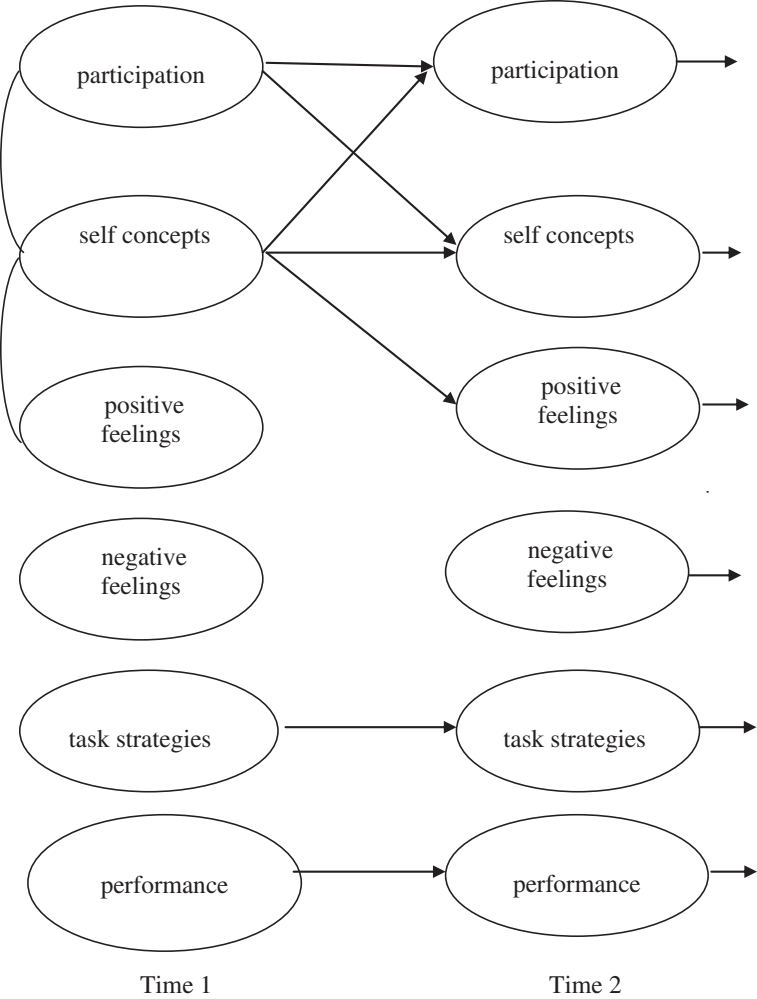

Supplement: Supplementary file 5 — Authors’ original file for figure 5 [file 40064_2013_619_MOESM5_ESM.pdf]

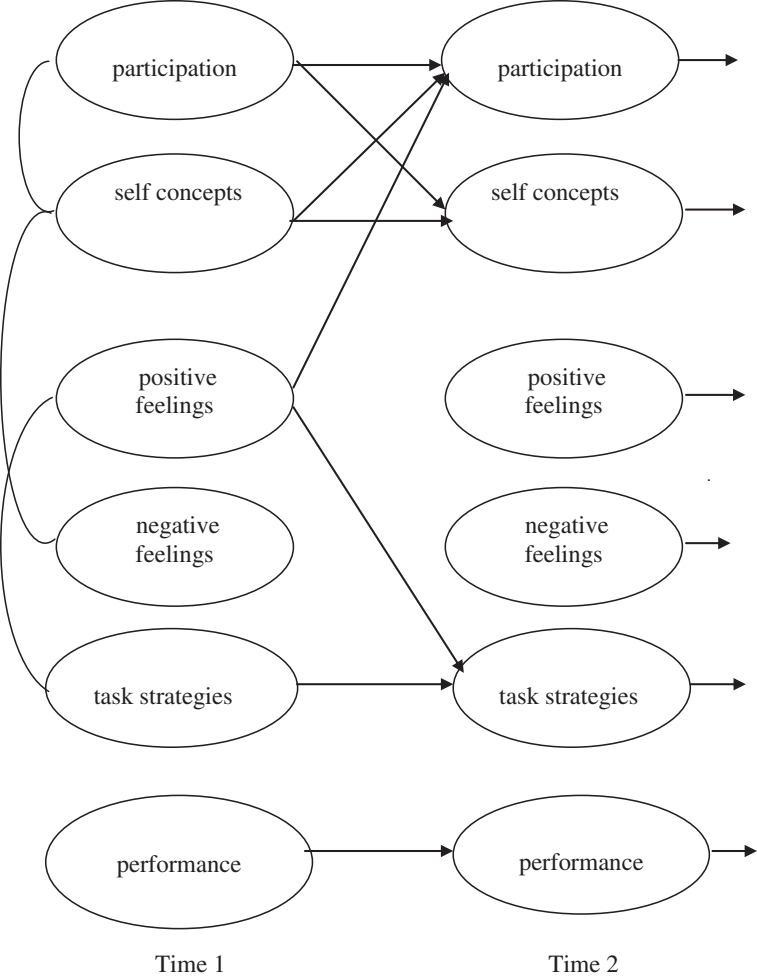

Supplement: Supplementary file 6 — Authors’ original file for figure 6 [file 40064_2013_619_MOESM6_ESM.pdf]
