# Supplementary material for: Motivational Spiral Models (MSM): common and distinct motivations in context
Source: Springerplus. 2013 Oct 25;2(1):565. doi: 10.1186/2193-1801-2-565 (PMC3825061; doi:10.1186/2193-1801-2-565)
Supplement: Supplementary file 2 — Authors’ original file for figure 2 [file 40064_2013_619_MOESM2_ESM.pdf]

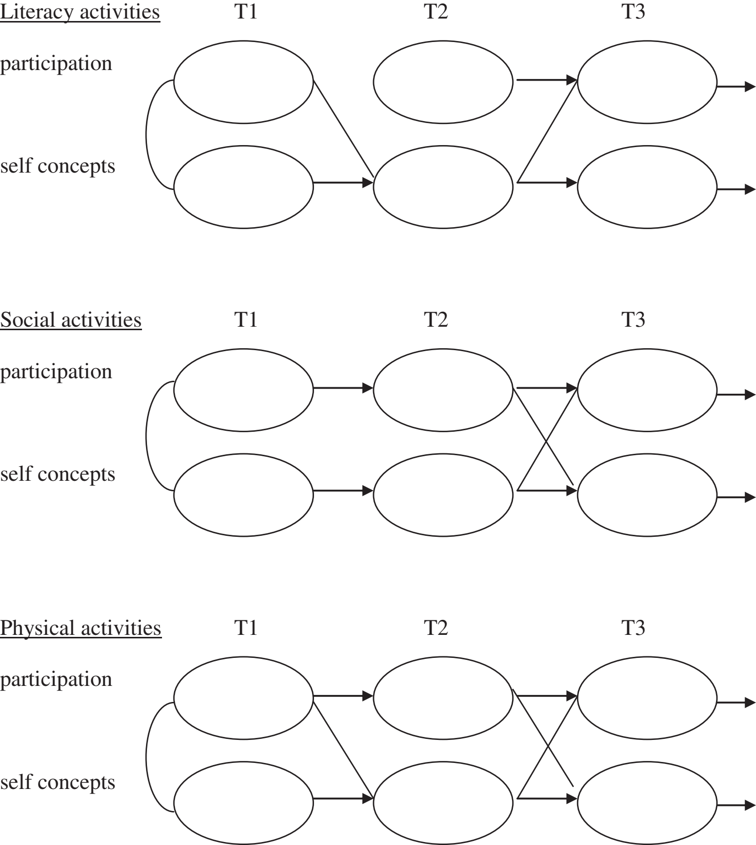
Literacy activities
T1
T2
T3
participation
self concepts
Social activities
T1
T2
T3
participation
self concepts
Physical activities
T1
T2
T3
participation
self concepts
