# Supplementary material for: Motivational Spiral Models (MSM): common and distinct motivations in context
Source: Springerplus. 2013 Oct 25;2(1):565. doi: 10.1186/2193-1801-2-565 (PMC3825061; doi:10.1186/2193-1801-2-565)
Supplement: Supplementary file 7 — Authors’ original file for figure 7 [file 40064_2013_619_MOESM7_ESM.doc]

Table 1.

Profiles and development of self concepts and participation over time (a month apart)

_______________________________________________________________________

Context means (sd) Time 1 Time 2 Time 3

_______________________________________________________________________

Literacy self concepts 3.88 (0.89) 4.05 (0.83) 4.03 (0.91)

participation 4.22 (0.83) 3.89 (1.18) 4.04 (1.07)

Social self concepts 4.13 (1.02) 4.21 (0.92) 4.13 (0.96)

participation 4.25 (1.02) 4.30 (0.99) 3.92 (1.36)

Physical self concepts 3.93 (1.22) 4.47 (0.61) 4.31 (1.03)

participation 4.34 (1.15) 4.52 (0.87) 4.21 (1.07)

________________________________________________________________________

Context regression (β) p<.05 Time (1 to 2) Time (2 to 3)

_______________________________________________________________________

Literacy self concepts 0.32 0.27

participation ns 0.52

self concepts:participation ns 0.25

participation: self concepts 0.38 0.20

Social self concepts 0.86 0.58

participation 0.51 0.33

self concepts:participation ns 0.48

participation: self concepts ns 0.28

Physical self concepts 0.25 0.57

participation 0.52 0.36

self concepts:participation ns 0.35

participation: self concepts 0.35 0.25

_______________________________________________________________________

Table 2.

Profiles for Motivational Spiral Models of Literacy, Social and Physical activities,

with reliability, mean (sd) and range, at Time 1 and Time 2 (a year later)

_______________________________________________________________________

Motivational Spiral Models Time 1 Time 2

alpha mean (sd) mean (sd)

_______________________________________________________________________

MSM-L Literacy skills ^a^ .79 3.1 (0.9) 3.3 (0.7)

task strategies .73 3.2 (0.9) 3.4 (1.0)

self concepts .86 3.8 (1.1) 3.9 (1.1)

positive feelings .69 3.7 (1.6) 3.3 (1.3)

negative feelings .83 1.6 (0.9) 1.3 (0.5)

participation .77 3.7 (1.5) 3.7 (1.4)

MSM-S Social skills ^a^ .90 4.0 (0.9) 4.7 (0.6)

task strategies .83 3.1 (1.0) 3.4 (0.9)

self concepts .68 4.3 (0.7) 4.2 (0.9)

positive feelings .67 3.6 (1.5) 3.1 (1.3)

negative feelings .66 1.7 (0.8) 1.4 (0.5)

participation .71 4.2 (1.2) 4.4 (1.0)

MSM-P Physical skills ^a^ .70 3.8 (0.7) 4.2 (0.9)

task strategies .83 3.1 (1.1) 3.5 (0.9)

self concepts .86 4.4 (0.9) 4.3 (0.8)

positive feelings .71 3.8 (1.4) 3.6 (1.2)

negative feelings .90 1.4 (0.9) 1.2 (0.5)

participation .77 4.5 (0.9) 4.4 (1.1)

_____________________________________________________________________

Note. a. for ease of comparison the skills scores are re-scaled from (1) low to (5) high

Table 3.

Profiles for clusters^a^ of children in MSM pathways (Time 1 to Time 2) for skills, self concepts, feelings, strategies and participation in literacy, social and physical activities.

______________________________________________________________________

MSM pathways Cluster A Cluster B

Profiles Time1 Time2 Time1 Time2

_______________________________________________________________________

MSM-L Literacy Cluster A (38%) Cluster B (62%)

skills ^b^ 3.1 3.2 3.2 3.4

task strategies 1.0 1.1 3.8 4.5

self concepts 1.4 1.8 4.4 4.8

positive feelings 3.7 2.3 5.0 5.0

negative feelings 1.7 1.0 5.0 2.0

participation 1.0 2.0 5.0 5.0

MSM-S Social Cluster A (18%) Cluster B (82%)

skills ^b^ 3.5 4.4 4.0 4.7

task strategies 3.9 2.5 2.7 3.8

self concepts 4.0 3.2 4.8 5.0

positive feelings 2.3 1.0 5.0 5.0

negative feelings 1.3 1.3 2.0 1.3

participation 1.0 5.0 1.0 5.0

MSM-Physical Cluster A (18%) Cluster B (82%)

skills ^b^ 3.7 4.0 4.0 4.5

task strategies 3.5 5.0 1.0 4.2

self concepts 3.2 2.6 5.0 5.0

positive feelings 5.0 1.0 2.3 5.0

negative feelings 5.0 2.3 1.3 1.0

participation 3.0 1.0 2.0 5.0

_____________________________________________________________________

Notes.

a. Cluster analysis used ‘K-means’ non-hierarchical method (e.g., Aunola et al, 2002).

b. For ease of comparison skills scores are re-scaled to range from 1 (low) to 5 (high).
